# Under What Conditions Can Electricity Be of Positive Service to the Gynecologist?

**Published:** 1891-03

**Authors:** Andrew H. Currier

**Affiliations:** New York


### UNDER WHAT CONDITIONS CAN ELECTRICITY BE OF POSITIVE SERVICE TO THE GYNECOLOGIST?

Read before the Section of Obstetrics of the Academy of Medicine, New York, Jan. 1891.

BY ANDREW H. CURRIER, M. D., NEW YORK.

The testimony upon this subject is conflicting. Some have opposed it from prejudice and bias, and others have advocated it with an enthusiasm which revealed indiscretion and unwisdom. Satisfactory knowledge can be gained only by experience, and this necessitates no little expense for the apparatus, and time and labor in order to comprehend the physical laws governing electricity. As in religion, science, art and politics, success only comes as a rule to those who follow up the subject persistently and thoroughly. The patient also must submit to such conditions as will permit of a fair test of the agent. The subject is considered under three headings :

1. Necessary outlay and apparatus.
2. Indications.
3. Contra-indications, cautions and objections.

The faradic current is indicated when one desires increased muscular tone or contractile force. Incidentally will come improved vascularity and nerve energy. The galvanic current is indicated as an astringent, hæmostatic, denutrient, admutrient or sedative. For some conditions, for example, pain, either current may be effective. All battery currents are based upon Ohm's law, that is the available battery force equals the entire force generated by all the cells, divided by the resistance offered by the wires, the fluid in the cells, in fact everything which hinders the passage of the current. The unit of usable current in electro-therapeutics is the milliampere. The requirements for a faradic battery are that it is small, simple, clean and cheap. Gaiffe's costs but a few dollars and is perhaps the best there is.

The requirements for a galvanic battery are steadiness of current, cleanliness, simplicity of construction and durability. The writer has never found a portable battery which answered these requirements, but does not assert that they do not exist. To answer the conditions mentioned there should be a large number of large cells in continuous connection. Either the Law or the Leclanche cells will give satisfaction, the former being more cleanly and more durable. A rheostat and a milliampere-meter are indispensable, and the writer is well pleased with the Bailey rheostat and the Barret meter graduated to 250. The connecting cords from battery to patient should be long enough that the patient may be moved about without danger of breaking circuit and giving shock. For an abdominal electrode, Martin's is the best. There are many varieties of vaginal and uterine electrodes, those designated by Apostoli being very good ones. The writer has designed one of Alluminium with a cylindrical removable platinum tip, the shaft being covered with thin rubber tubing. It is light, cheap and flexible. The rheostat and meter may rest upon portable base, furnished with suitable bending posts and switch for changing polarity. The character and effect of the current at the two poles is essentially different. The positive pole will check hemorrhage and glandular secretion, the negative will not. The positive pole will corrode all but the noble metals, the negative will not. The positive pole is acid, the negative alkaline; at the positive pole oxygen is liberated in the electrolysis of water, at the negative hydrogen.

The writer's paper contains analysis of 23 cases in which the indications for treatment were:

1. Pain.
2. Hemorrhage.
3. Inflammatory exudate.
4. Sterility.
5. Dysmenorrhœa.
6. Super-secretion.
7. Hysteria.
8. Uterine subinvolution.
9. Uterine sub-nutrition.

For pain the positive pole should be within the vagina or uterus, and a weak current is better than a strong one. A good average is 30 milliamperes, used from five to eight minutes. The intervals of application should depend upon the duration of the periods in which pain is absent. Pain was relieved in two cases in which it persisted after removal of the uterine adnexa, in one each of uterine myoma, pro-salpinx with ovarian apoplexy, and endometritis, and two of pelvic peritonitis, with exudation. For hemorrhage the positive pole is believed to be unsurpassed. It was used in a case of interstitial myoma, and in one of malignant disease of the uterus and omentum. Four cases were treated for inflammatory exudate, and in three the exudate was disintegrated and absorbed. But as the diseased organs which had been confined by it became more mobile they also became larger and more sensitive. In five cases sterility was treated with the faradic current. Impregnation and delivery resulted in two. Dysmenorrhœa may be relieved by either the positive galvanic pole or by faradism. Three cases are narrated, but in only one was the result decidedly favorable. For super-secretion the positive pole is preferable to the powerful caustics and escharotics, and yielded good results in three cases. In two cases hysterical symptoms were much modified in addition to benefit which was derived form more palpable lesions.

Subinvolution was successfully treated in one case, the uterus contracting firmly upon the bi-polar electrode of Apostoli, and with the faradic current. Uterine sub-nutrition in connection with hard anteflexed uteri, and usually associated with amenorrhœa, dysmenorrhœa or sterility, will be benefited by the faradic current. Five cases were treated, and all but one received positive benefit. Under the head of cautions, contra-indications and objections, nausea resulted in one case, and this observation has frequently been made by others. The passage of the galvanic current may cause faintness, which may be slight or profound, and dizziness. In a case of exopthalmic goitre with rapid heart action, collapse was imminent on two occasions. An irritable heart, such as is usually present in the last mentioned disease, and with certain chronic gastric disorders contra-indicates the use of electricity. Malignant disease within the abdomen is a contra-indication, or at least proved so in one case. Small, dry electrodes should not be applied to the abdomen, but large, wet ones. The former will invariably produce burning. The method of rapid reversals of the galvanic current is of limited usefulness, and should not be used with nervous women. The shocks may be exceedingly harmful. The electro-puncture of fibroid tumors means possible sepsis, with its consequences. If it is electricity, and not inflammation and sloughing which reduce the nutrition of a tumor, it would seem to be unnecessary. Galvano-cauterization of the uterine mucous membrane seems to furnish the advantage of puncture without danger. Electro-puncture is also disapproved for haematoma and haemotocele as dangerous, tedious and inefficient as to its results. Electricity is the handmaid, and not the mistress of surgery, a valuable assistant and increasing in value with experience, but one which demands rational, careful and intelligent use.

